# Emergence of COVID-19 Variants: An Update

**DOI:** 10.7759/cureus.41295

**Published:** 2023-07-03

**Authors:** Parakriti Gupta, Varsha Gupta, Chander Mohan Singh, Lipika Singhal

**Affiliations:** 1 Microbiology, Government Medical College and Hospital, Chandigarh, Chandigarh, IND

**Keywords:** antivirals, vaccines, immune escape, variants, covid-19

## Abstract

Severe acute respiratory disease virus-2 (SARS CoV-2) is one of the deadliest global threats faced by mankind to date. Despite the colossal efforts, the viral pandemic swept across all boundaries. Besides the virulence and susceptible population, the low proofreading capacity and error-prone mechanism of RNA-dependent RNA polymerase (RdRp) have contributed to new variants and reinfections. The World Health Organization has officially categorized these variants as variants of concern or variants of interest. This nomenclature is not merely to suffice the surveillance but also to have effective treatment and vaccine options in place. Coronavirus disease 2019 (COVID-19) variants have the propensity to render the available treatment strategies futile owing to the mutations they acquire. The futility of treatment strategies can be attributed either to the ineffectiveness or the shortage of supply given the skyrocketing increase in the number of cases. Presently, the Omicron variant is the most widespread one and is known to escape the protection, be it immune-derived, vaccination-derived, or hybrid. WHO has recommended modification in vaccine development policies and few companies have introduced Omicron-adapted vaccine jabs. Keeping in view the unending tale of COVID-19 variants and the huge data available on the same, this review focuses on providing insight into the emergence and ongoing dynamics of these new COVID-19 variants.

## Introduction and background

Severe acute respiratory disease virus-2 (SARS-CoV-2) is one of the most dreadful threats to mankind and has culminated into an unrelenting, fatal pandemic. The virus emerged in Wuhan, China, in late 2019 affecting a cluster of patients with pneumonia of unknown etiology [1-4]. Consequently, the infection was affirmed as a ‘public health emergency of international concern’ on January 30, 2020, by the World Health Organization (WHO) [1,3,4]. The origin of the virus was traced back to the wet live markets of China, which facilitated the zoonotic transmission of disease by spillover across the animal-human interface. The virus gradually gripped the whole world, and WHO acknowledged it as a global pandemic on March 11, 2020 [1-4]. Genome sequencing of the agent unraveled the virus to be a member of the order Nidovirales, family *Coronaviridae*, and genus *Betacoronavirus* of 2B lineage. The phylogeny of the virus was also tracked down to the SARS-like bat betacoronavirus, RaTG13. SARS-CoV-2 is a non-segmented, positive-sense, single-stranded, enveloped virus with the potential to infect mammals and birds. The virus causes mild-to-severe respiratory illness (most common), followed by gastrointestinal illness. It was designated as coronavirus disease 2019 (COVID-19) by WHO [1-4]. Its dreadful transmission has not halted since its emergence and infection keeps on waning and waxing, giving rise to new waves as soon as the herd immunity depletes or new variants arise.

## Review

Mutations in SARS-CoV-2 and origin of variants

SARS-CoV-2, being an RNA virus, has the propensity to mutate due to its low proofreading capacity and error-prone mechanism of the RNA-dependent RNA polymerase (RdRp), also known as non-structural protein (Nsp) 12 [3,4]. However, unlike the influenza virus, SARS-CoV-2 is non-segmented. There is no antigenic drift/shift seen in COVID-19. SARS-CoV-2 undergoes constant mutations and amino acid substitutions in its genome, and even a single amino acid substitution drastically influences the virulence and transmission ability of the virus. However, all mutations are not that pronounced and may remain silent with minor changes in phenotypic characteristics. The non-silent, aggressive mutations are the predecessors of new variants [5].

Variants are viruses that belong to the same class but have varied and mutated genome sequences. They are either more aggressive or milder than the parent virus. Variants harbor single or multiple mutations. Once these variants acquire distinct physical properties from the parent virus, it is referred to as a strain. All strains are variants but not vice-versa [6].

These mutations occur recurrently in the spike protein of the genome. The SARS-CoV-2 genome (27-32kb) consists of 16 Nsps (Nsp 1-16) and four structural proteins including envelope (E), membrane (M), spike (S), and nucleocapsid (N). Nsp is involved in replication, translation, and signaling pathways. Nsp12 encompasses RdRp, the most critical initiator of mutations. Among the structural proteins, the S glycoprotein has two subunits S1 and S2, which facilitate the entry of the virus into host cells. S1 has an N-terminal and a receptor binding domain, which helps in binding to host receptors, angiotensin-converting enzyme 2 (ACE 2). The S2 subunit fuses the viral membrane with host cells. It consists of a cytoplasmic tail, a fusion peptide, a transmembrane domain, heptad repeat 1 and 2, a connector domain, and a central helix. The furin-cleavage site is located at the boundary of the S1/S2 subunit with four residues [7,8]. The S glycoprotein is the most important factor that determines the origin of variants and strains, during mutations. The first significant mutation of COVID-19 in the S protein was D614G, which had the same severity and increased transmissibility compared to the ancestral strain. The D614G mutation is present in >98% of SARS-CoV-2 strains. After the emergence of this mutation, numerous mutations have been reported in the genome of SARS-CoV-2, making the virus more pathogenic, thus resulting in its continuous transmission. Natural selection determines the survival or culling of various mutations. The ones with enhanced fitness and competitive survival, along with increased transmission, virulence, or immune escape are prone to increased survival and vice versa [8]. The founder effect also shapes the viral evolution, by allowing a limited viral population with characteristics compromising the viral fitness to dominate as a separate viral populace [9].

Nomenclature of emerging variants

Since a wide plethora of variants keep on originating and vanishing, the naming of these variants is pertinent. Global Initiative on Sharing Avian Influenza Data (GISAID), Phylogenetic Assignment of Named Global Outbreak (PANGO), and Nextstrain have been assigned to analyze, curate, and make the genome sequences available for open access. Of these, GISAID was established in 2008 for making genomic data of influenza virus available in open access. After the declaration of the COVID-19 pandemic in Jan 2020, GISAID is dealing with SARS-CoV-2 as well [10]. PANGO is a software-based lineage nomenclature tool, developed by the Andrew Rambaut Lab and the Centre for Genomic Pathogen Surveillance in South Cambridgeshire, United Kingdom [11]. Nextstrain is a rather new platform under the collaboration of researchers in Seattle, United States, and Basel, Switzerland, that facilitates the comprehension of viral evolution and transmission [12]. Apart from these global nomenclature systems, national or institute-wise nomenclature can also be adopted.

Ever since the emergence of the D614G mutation, the SARS-CoV-2 genome has undergone many deletions, substitutions, and insertions. Some reports state that SARS-CoV-2 acquires one to two mutations per month [13,14]. All these genomic events are supervised by the Technical Advisory Group on Virus Evolution (TAG-VE), which was previously designated as WHO Virus Evolution Working Group. These working groups have come up with non-stigmatizing labels for variants using Greek letters for easy comprehension by the general public viz. alpha, beta, delta, and the like. It was the emergence of a wide array of variants in the later months of 2020 that impelled the molecular description and characterization of varied SARS-CoV-2 variants as ‘Variants of Interest’ (VOIs) and ‘Variants of Concern’ (VOCs). It was of substantial importance to underline such prioritization for global monitoring, surveillance, and research efforts [13].

The group of closely related viruses with a common ancestor is referred to as ‘lineage’ and the emerging variants are further categorized based on viral characteristics. The working definition of all these variants is changed in real-time based on changes in transmission, severity, immune escape, and impact on diagnostics and vaccination [13].

VOI

As per the working definition given by WHO, a VOI is a SARS-CoV-2 variant that possesses “genetic changes predicted or known to affect its characteristics like transmissibility, disease severity, immune escape, diagnostic or therapeutic escape. They have been identified to cause significant community transmission or multiple COVID-19 clusters, in multiple countries with increasing relative prevalence with the increasing number of cases over time, or other apparent epidemiological impacts to suggest an emerging risk to global public health" [15]. WHO has designated XBB.1.5 (11-01-2023) and XBB.1.16 (17-04-2023) as VOI, as of June 27, 2023 [15]. However, currently there are no VOI, as per CDC [13]. The previous VOIs have been tabulated in Table 1.

**Table 1:** Nomenclature, country of origin, date of declaration, and Pangolin lineage of VOIs as of June 2022 VOI: variants of interest Source: World Health Organization [15]

WHO Label	Country of origin	Date of declaration as VOI	Date of declaration as Previous VOI	Pangolin lineage
Epsilon	United States	March 5, 2021	July 6, 2021	B.1.427, B.1.429
Zeta	Brazil	March 17, 2021	July 6, 2021	P.2
Eta	Multiple	March 17, 2021	September 20, 2021	B.1.525
Theta	Philippines	March 24, 2021	July 6, 2021	P.3
Iota	United States	March 24, 2021	September 20, 2021	B.1.526
Kappa	India	April 4, 2021	September 20, 2021	B.1.617.1
Lambda	Peru	June 14, 2021	March 9, 2022	C.37
Mu	Colombia	August 30, 2021	March 9, 2022	B.1.621

VOC

As per the working definition given by WHO, a VOC is a SARS-CoV-2 variant that meets the definition of “VOI and after thorough comparative assessment, has been noted to cause an increase in transmissibility, a detrimental change in COVID-19 epidemiology, an increase in virulence, change in clinical disease presentation, or a decrease in the effectiveness of public health and social measures or available diagnostics, vaccines, and therapeutics" [15]. Apart from Omicron, four other variants that wreaked havoc earlier have now been designated as ‘previous VOCs’ (Table 2).

**Table 2:** Nomenclature, country of origin, date of declaration, and Pangolin lineage of ‘previous VOCs' as of June 2022 VOC: variants of concern Source: World Health Organisation [15]

WHO Label	Country of origin	Date of declaration as VOC	Date of declaration as Previous VOC	Pangolin lineage
Alpha	United Kingdom	December 18, 2020	March 9, 2022	B.1.1.7
Beta	South Africa	December 18, 2020	March 9, 2022	B.1.351
Gamma	Brazil	January 11, 2021	March 9, 2022	P.1
Delta	India	April 4, 2021 (VOI: May 11, 2021)	June 7, 2022	B.1.617.2
Omicron	South Africa	November 21, 2021	Newer emerging sublineages dominating the world	B.1.1.529

VOC Lineages Under Monitoring (VOC-LUM)

In May 2022, the WHO added another category of “potentially concerning sub lineages” of widespread VOCs, and designated them as “VOC lineages under monitoring (VOC-LUMs).” This new category was devised to supervise the intra-VOC evolution. It has evolved with the emergence of a constellation of descendent lineages since the emergence of Omicron. Whenever any of these lineages is proven to have characteristics distinct from the original VOC, it will be designated a separate label by TAG-VE [15,16].

As per the working definition given by WHO, VOC-LUM is a SARS-CoV-2 variant that belongs to a currently circulating VOC, based on the phylogenetic analysis, which shows signals of transmission advantage compared to other circulating VOC lineages, and has additional amino acid changes known or suspected to confer the observed change in epidemiology and fitness advantage as compared to other circulating variants [15,16] (Table 3).

**Table 3:** Country of origin and Pangolin lineage of VOC-LUM as of June 2022 VOC: variants of concern; LUM: lineages under monitoring

Pangolin lineage	Country of origin	Relationship to VOC
BA.4	South Africa	Sister lineages: BA.1 and BA.2
BA.5	South Africa	Sister lineages: BA.1 and BA.2
BA.2.12.1	United States	BA.2 sublineage
BA.2.9.1	Multiple	BA.2 sublineage
BA.2.11	Multiple	BA.2 sublineage
BA.2.13	Multiple	BA.2 sublineage
BA.2.75	India	BA.2 sublineage

Variants Under Monitoring (VUM)

As per the working definition given by WHO, VUM is a SARS-CoV-2 variant that has undergone genetic changes suspected to impact the viral characteristics that might pose some future risk, but the evidence of epidemiological or phenotypic impact is currently unclear and needs enhanced monitoring and repeated assessments. VUM can be designated as VOC or VOI with evolving evidence on a real-time basis by WHO [15,16]. WHO has issued the statement pertaining to monitoring of sublineages of Omicron and decided to consider their classification independently, as VUM or VOI or VOC; from March 15, 2023 [15]. The present VUM are depicted inTable 4.

**Table 4:** Country of origin and Pangolin lineage of VUM, as of June 19, 2023 ? indicate that the origin of certain variants isn't clear VUM: variants under monitoring

Pangolin lineage	Country of origin	Derivation
BA.2.75	India	BA.2 sublineage
CH.1.1	Southeast Asia	BA.2 sublineage
XBB	?Singapore/?India	BA.2 sublineage: Recombinant of BA.2.10.1 and BA.2.75 sublineages (BJ1 and BM.1.1.1)
XBB.1.9.1	Southeast Asia/?Singapore/Indonesia	BA.2 sublineage: Recombinant of BA.2.10.1 and BA.2.75 sublineages (BJ1 and BM.1.1.1)
XBB.1.9.2	?Southeast Asia/?Singapore/Indonesia	BA.2 sublineage: Recombinant of BA.2.10.1 and BA.2.75 sublineages (BJ1 and BM.1.1.1)
XBB.2.3	India/?United States	BA.2 sublineage; Recombinant of BA.2.10.1 and BA.2.75 sublineages (BJ1 and BM.1.1.1)

Variants of High Consequence (VOHC)

As per the working definition given by the CDC and SARS-CoV-2 Interagency Group (SIG), VOHC is a SARS-CoV-2 variant with clear evidence of decreased effectiveness of preventive or medical countermeasures, as compared to the prior variants in circulations [5]. VOHC possesses characteristics, in addition to those present in VOC, like a failure in diagnostic procedures, evidence to suggest a substantial decrease in the effectiveness of the vaccine, a higher rate of vaccine breakthrough infections or very low vaccine-induced protection during severe disease, a substantial reduction in susceptibility to approved drugs, and an increased severity of clinical infection with more hospitalizations. No SARS-CoV-2 variant has been designated as VOHC till now [13].

Some important variants

Alpha Variant (B.1.1.7)

This was the first SARS-CoV-2 to be designated as a VOC. It was first detected in the United Kingdom, in October 2020. The variant was peculiar in having 23 more mutations than the Wuhan strain, eight being in the S protein, and was responsible for the surge in cases in the United Kingdom. The alpha variant was considered to have an escalated (40-80%) transmission, attributed partly to N501Y, P681H, and 69/70 deletions. The mutations were initially considered to increase the virulence; however, later studies deferred the hypothesis [17]. The WHO de-escalated the alpha variant and its sub-variants to ‘previously circulating VOC’ in March 2022 [15].

B.1.1.7 with E484K

This variant is characterized by an additional E484K mutation and is also known as 'VOC 21FEB-02' or B.1.1.7 with E484K [18]. This mutation in spike protein decreases the neutralization efficiency with polyclonal antibodies and post-vaccination sera. It has been considered an escape mutation as it helps the virus to escape easily from the body’s immune system [19].

Beta Variant (B.1.351)

The B.1.351 variant was first detected in Nelson Mandela Bay in South Africa in October 2020 [17]. It is also referred to as 501Y.V2 and possesses 23 mutations with 17 amino acid changes. The most significant of these include E484K, N501Y, and K417N mutations in the S protein. The notable features of this variant were the presence of escape mutation under E484K and K417N, its escalated transmissibility, and comparatively common infection of young immunocompetent individuals [17,20], mediating escape from antibody neutralization and decreased effectiveness of vaccines [21].

Gamma Variant (B.1.1.28.1 or P.1)

The B.1.1.28.1 or P.1 variant was initially detected in Manaus in the Amazonas state of North Brazil [21]. It was noted to have 17 amino acid substitutions, 10 being in S proteins and the key mutations being N501Y, E484K, and K417T in the receptor binding domain (RBD) that enhanced its affinity to the human ACE-2 receptor. The immune escape mutation was attributed primarily to E484K. The P1 variant was noted to be 2.5 times more transmissible than the original coronavirus. The variant has shown relative resistance to neutralization by convalescent plasma and vaccination [22].

Kappa Variant (B.1.617.1)

B.1.617.1 was first detected in India in December 2020. This variant is one of the three sublineages of B.1.617 [23]. Public Health England designated it as a variant under investigation (VUI-21APR-01) [24]. The important mutations noted in this variant were L452R, E484Q, and P681R [25].

Delta Variant (B.1.617.2)

The B.1.617.2 variant was initially detected in Maharashtra, India in late 2020/early 2021. Since then, it has spread across the world and has been reported from more than 135 countries. The delta variant was of paramount concern as the variant was noted to be more transmissible (1.1-1.4 folds) than the alpha and kappa variants [26]. It possesses mutations in the S protein with T478K, P681R, and L452R being the most prominent ones. This variant is known to affect the transmissibility and neutralization of antibodies. This variant is considered to be one of the most transmissible respiratory viruses known and accounted for heightened mortality [27].

Delta Plus Variant (Delta With K417N)

This variant originated from the delta variant, after the acquisition of the K417N mutation. It was first reported in June 2021. This mutation was noted in previous beta and gamma variants and was feared to have reduced vaccine effectiveness and neutralization with antibodies. There were also concerns about the increased risk of reinfection. This Delta with K417N variant has two clades that correspond to the AY.1 and AY.2e Pango lineages, respectively [18,27].

Omicron Variant

The Omicron variant was first reported in November 2021 in South Africa. This is considered the most mutated variant, with approximately 50 mutations in its genome, of which 26-32 mutations are in the S protein. The most worrisome feature of these mutations is that 15 of them are in the RBD (receptor binding domain) [28,29]. These numerous mutations have led to an increase in the transmissibility of this variant along with the immune escape from hybrid, natural and vaccine-induced immunity [28,30]. These have rendered the use of convalescent serum and monoclonal/polyclonal antibodies ineffective [21,31]. The variant is characterized by the typical S gene dropout or S gene target failure (STGF), which is 69-70del. STGF has previously been associated with the alpha variant as well, but since the alpha variant is very rare these days, STGF is considered indirect evidence of omicron. However, the same needs to be confirmed by whole genome sequencing (WGS). Clinically, it has a shorter incubation period with milder symptoms and an increased risk of reinfection [32,33]. It has several sub-lineages, of which BA.1, BA.1.1, and BA.2 were the most commonly circulating ones, as of February 2022 [34]. Of these sub-lineages, BA.1 (21K) had 60 mutations, of which 32 are in the S protein, and BA.2 had 28 more mutations, four being in the S protein [35]. BA.2 (21L) is also referred to as the Stealth Omicron owing to the absence of the characteristic STGF in its genome. This absence of STGF and the presence of other confirmatory genes in PCR led to a few initial tribulations in discerning BA.2 and it was confused with the delta variant in PCR. However, the term Stealth is a misnomer, as BA.2 can be precisely determined and is considered more transmissible than BA.1. The standard sub-lineage is now referred to as BA.1 (B.1.1.529.1), while other sub-lineages are referred to as BA.2 (B.1.1.529.2), BA.3 (B.1.1.529.3) and so on [36]. BA.2 subvariants, BA.2.12 and BA.2.12.1 were also detected after a short while, with a substantial survival advantage over BA.2 [37,38].

Another overtaxing development was the origin of the XE subvariant, which is a recombinant lineage of BA.1 and BA.2. XE subvariant was isolated firstly from the United Kingdom in January 2022 and studies found it to be more transmissible than BA.1 and BA.2, designating it as the most contagious variant identified till then [39]. BA.3, the third Omicron sublineage, is quite rare. However, more recently, the two most worrisome subvariants, BA.4 and BA.5, have emerged globally. BA.4 was detected for the first time in South Africa in January 2022, followed by BA.5, but the widespread transmission started in April 2022, leading to a newer COVID-19 wave in many countries [40]. BA.4 and BA.5 are more similar to BA.2 while retaining STGF (69-70del) from the BA.1. Both BA.4 and BA.5 are similar to each other in the S region and vary merely outside the S region. These variants can escape the immune system efficiently, by acquiring mutations in the S protein, L452R, F486V, and Q493 [41]. These were classified as VOCs by the European CDC and the UK Health Security Agency in May [42,43]. Presently, these are the most common SARS-CoV-2 variants circulating globally, being responsible for fresh COVID-19 waves [44-46].

Recently, BA.2.75 has been reported to have emerged as a new circulating variant. BA.2.75 was first reported in India in May this year and subsequently, has been identified from approximately 10 countries. The variant is supposed to be more transmissible with more ability of immune escape than other Omicron subvariants; however, the data isn’t enough as of now, and WHO hasn’t declared it as VOI or VUM till now. However, ECDC has declared BA.2.75 as VUM [47].

Another new variant of BA.5, BF.7, has also been reported lately. This variant is noted to have more transmissibility, with a shorter incubation period and more chances of re-infection [48]. Apart from these, another offshoot variant, XBB.1.5, is now being considered the one determined to spread globally. XBB.1.5 is known to have multiple mutations in the spike protein, with a rare amino acid change named F486P, potentiating its transmissibility and immune escape [49].

Surveillance and monitoring of SARS-CoV-2 variants

SIG, in coordination with CDC, WHO, National Institute of Health (NIH), Food and Drugs Administration (FDA), Biomedical Advanced Research and Development Authority (BARDA), and other nations as well as other international health agencies, is monitoring the waxing and waning of COVID-19 variants on a real-time basis [13]. Indian SARS-CoV-2 Genomics Consortium (INSACOG) is monitoring the situation in India, in collaboration with the Department of Biotechnology and the Indian Council of Medical Research [50]. These along with their counterparts from around the world are using WGS as the platform to decipher the circulating strains, so as prioritize the potentially important mutations and strategize preventive and management strategies accordingly. Integrated genomic surveillance systems across the globe are of paramount significance in the documentation and control of SARS-CoV-2 in national as well as international settings as this genomic data can be studied in real-time to predict and rule out any impending, potential strains of the pandemic.

Impact of variants on available treatment strategies

COVID-19 variants have the propensity to render the available treatment strategies futile, because of the mutations they acquire. The futility of treatment strategies can be attributed either to the ineffectiveness or indirectly to the shortage of supply due to skyrocketing increase in the number of cases. It was noted that the number of mutations increased from two per month to several folds, especially in the N-terminal and the receptor binding domain, thereby, lessening the efficacy of treatment modalities [51]. The most substantial D614G mutation twirled the virus into a much more infective variant by increasing its affinity to ACE2 receptors and consequently, the entry into infective cells. N439K, the successive mutation of D614G, commanded a noteworthy lineage B.1.258 with enhanced binding affinity and reduced neutralizing action of monoclonal antibodies and polyclonal ones from convalescent sera of infected patients [52]. However, the deep mutational scanning study didn’t have the same findings, and the discordance has been attributed to the mechanism of immune escape exhibited by this mutation [51]. The N439K alters immune evasion by enhanced affinity to ACE-2 receptors, rather than altering the epitope recognition, thereby giving inconsistent results. Another mutation that provides an immune escape to the virus is E484K, which was reported in the South African B.1.351 and Brazilian B.1.1.28 strains initially [51,53]. This mutation was responsible for the ineffectiveness of Bamlanivimab in COVID-19 patients because of non-neutralization, especially in patients infected with the B.1.1.7 variant [53]. Another mutation, S477G in the S protein, has been noted to confer immune escape from monoclonal antibodies, but not from convalescent sera [51]. Other mutations conferring reduced binding with monoclonal antibodies and associated with immune escape include S: L18F, S: Y144-, and S: K417 [54-56].

The most dreadful VOC is Omicron, which is taking a toll on global health with >30 mutations in the S protein, 15 being in the RBD region. Omicron possesses T478K, K417N, E484A, N501Y, N440K, Y505H, S477N, Q493R, G496S, and Q498R mutations; and deletions in orf1a i.e., L3674, G3676, and S3675 and deletions in orf9b i.e., E27, A29, and N28. These mutations are responsible for immune escape, in addition to increased transmissibility. Studies have revealed that neutralization antibody titre was 36 times lower when convalescent sera from patients infected with previous strains was neutralised with pseudotyped Omicron S protein-expressing virus and the same was 39 times lower with Delta strain [28]. BA.4 and BA.5 are thought to have originated from BA.2 but carry unique mutations such as L452R and F486V in the S protein, which might be conferring escalated transmissibility and immune evasion [57,58]. The authors studied the effect of Bamlanivimab on P.1 and B.1.351 variants and a full immune escape in these variants was unraveled [59]. On the contrary, partial immune escape with Casirivimab was noted [59]. Another study disclosed the loss of efficacy of antibody cocktails in variants possessing novel mutations in spike and RBD region and implicated that the most common mutations responsible for this immune escape were E484K, L452R, and K417N/T [60,61].

Impact of variants on the effectiveness of vaccines

The vaccines have been running an unending taxing race against the continuously evolving SARS-CoV-2 variants, since its emergence [40]. Though none of the VOCs have rendered vaccines fully futile, the effectiveness of these vaccines has reduced modestly over time, for the newly emerging variants [51-54,62-66]. Despite the reduced effectiveness, vaccines do provide protection against severe disease and vaccinated individuals usually suffer from a milder infection. The vaccine vs disease wasn’t a substantial fear until the emergence of the delta variant [55]. Studies have shown that mRNA vaccines have better protection rates against delta variants as compared to the ChAdOx1 vaccines. Some studies have shown preserved T-cell recognition despite reduced antibody neutralization for the delta variant [55,62-66]. Multiple variations like L452R present in delta, iota, and epsilon variants have been noted to enhance virus infectivity and transmissibility, further reducing the neutralization ability of the vaccines and plasma [56,57]. On the other hand, the alpha variant does harbor increasing transmissibility of the mutations, but these don’t affect the binding of neutralizing antibodies. Similarly, beta and gamma variants are noted to be minimally affected by these mutations, with a mild decrease in the deterrence of symptomatic infection. However, one dose of ChAdOx1 or BNT162b2 is seen to have less efficacy in the delta variant, as compared to two doses of vaccination [67].

The Omicron variant is known to escape the protection, be it immune-derived, vaccination-derived, or hybrid. Studies have shown that vaccines have limited effectiveness against symptomatic Omicron infections. Moreover, the effectiveness after the booster doses waned faster [66]. Vaccine effectiveness for BA.1 and BA.2 was noted to be similar; however, the effectiveness for BA.2 waned faster as compared to BA.1 [64].To add to the near-ineffectiveness of prevailing vaccines, BA.4 and BA.5 have spread across the globe, affecting vaccinated individuals to a great extent. Lately, studies have shown a 4.4 times less effectiveness of vaccination against the BF.7 variant, as compared to the original strain [68]. Other subvariants like BA.2.75.2, BQ.1, and BQ.1.1 were noted to have more neutralization resistance against the vaccines. The same has been attributed to N460K and K4444 mutations in the latter, while F486S in the former. The results were confirmed using infectivity testing in Calu-3 cells and molecular modeling. 

Keeping in view these developments, Pfizer Inc. (New York, United States) and Moderna, Inc. (Massachusetts, United States) upgraded their vaccine development policies and have introduced Omicron-adapted vaccine jabs [69]. With the increasing number of affected individuals and the immune escape by new variants, the WHO has issued an interim statement regarding the use of variant-updated vaccines [70]. Its aim is the provision of greater protection against severity and fatality, simultaneously providing a broader horizon of protection for distant variants than the index virus. Moderna has developed a new bivalent mix-and-match vaccine based on the same terms, using two varied versions of spike proteins (the original and the changed version of BA.1). Data from the recent trial has shown 75% increased efficacy of this bivalent vaccine against BA.1 and three times lesser protection against BA.4 and BA.5 [71]. Though the results are encouraging, they are not quite compelling to shift to variant-based vaccines. The adoption of a pattern for seasonal influenza jab that is followed for influenza vaccines is still in the infancy for COVID-19 as the precise dynamics of evolution and imprinting are still cryptic. The regulatory authorities at WHO will review the data from these variant-adapted vaccines, and decide on the emergency use authorization of the same, which will be further considered for policy recommendations by Strategic Advisory Group of Experts on Immunization (SAGE).

## Conclusions

The emerging COVID-19 variants underline the efficacy of the virus in potentially escaping the immune system, simultaneously enhancing their entry, transmissibility, and lethality. The ongoing, continuous mutations in the error-prone SARS-CoV-2 genome render the diagnostic tests and susceptibility to treatment strategies futile. Mutations like N439K, E484K, S477G, L452R, F486S, L3674, G3676, and S3675 help the virus attain more infectivity and facilitate their immune escape. These dynamic changes further highlight the significance of adhering to preventive measures mask usage, hand hygiene, and isolation of suspected and confirmed cases, by both immunocompromised as well as immunocompetent individuals. Besides these, surveillance, genomic monitoring, determination of the circulating variants, and vaccination drive in the community still act as the key players in the control, mitigation, and prevention of this relentless pandemic.
